# Resuscitation of the Drowned

**Published:** 1886-06

**Authors:** 


### RESUSCITATION OF THE DROWNED.

The *Medical Advocate* mentions a case of drowning treated by certain physicians who had been in more or less active practice for over five years. They seemed entirely at a loss as to what to do first. They forgot all about how to produce artificial breathing, and what they did do was precisely what they ought not to have done, in half an hour they desisted, and pronounced the case hopeless. An ordinary boatman, who was, fortunately, experienced in such cases, arrived on the spot as the physicians ceased their efforts, and by proper management succeeded in saving a human life.

The instructions for resuscitating adopted and published by the United States Life Saving Service are so efficient, and at the same time so simple, that we commend them to the attention of the profession. They are, in substance, as follows: The moment the body is taken from the water it should be stripped to the waist. The clothing having been made into a roll to raise the pit of the stomach above the level of the mouth, all fluid should be forced out by pressure; this is done by placing one hand on the back, just below the scapulæ, and the other on the chest at a point directly opposite. Having proceeded thus far, with as little delay as possible, turn the body upon its back, place the roll of clothing under the small of the back, and practice artificial respiration as follows: Grasp the chest on either side of the pit of the stomach, gradually pressing forward and upward until the whole strength is used, and then suddenly letting go; continue to repeat this operation with the regularity of natural breathing. As there are cases on record where the respiratory function has been re-established after having ceased for more than an hour, it is well not to be impatient of results; any time within two hours your efforts may be crowned with success, without there being any previous sign to indicate it.

